# An empirical study of mainland Chinese students’ learning needs, perceptions of self-identity and sense of belonging

**DOI:** 10.1186/2193-1801-3-S1-O2

**Published:** 2014-12-04

**Authors:** Ricky Yuk-kwan Ng, Lai-fong Lau

**Affiliations:** Centre for Learning and Teaching, Vocational Training Council, Kragujevac, Hong Kong

## Background

This paper aims for an understanding of the learning needs of students from mainland China and their perceptions of self-identity and sense of belonging during their study in Hong Kong. Seeing the chances to promote internationalization, and with Hong Kong being one of the major Asian educational hubs, the Hong Kong government initiated higher education reform in early 2000 to widen the participation rate in higher education to cope with the acute demand for life-long learning in higher education programs in Hong Kong. The Sutherland Report on Higher Education in Hong Kong [1] suggested that the landscape of local higher education institutions be widened by strategically collaborating with overseas universities to develop new self-financed programs and provide additional places for incoming students. Subsequently, the government has encouraged local institutions to develop self-financed higher education programs to cater to the demand from local students and to recruit overseas students, especially those from mainland China. A survey of students from mainland China reveals their concerns about learning support, professional qualifications, daily living, social and cultural acceptance and economical and emotional adaptation during their study in Hong Kong [2]. Apparently, along with their cross-boundary learning experiences, students from mainland China are also confronting the struggles of localization and adaptation to the Hong Kong community. In view of the above, it would be beneficial to know the learning needs of mainland China’s students along with the learning and teaching strategies to accommodate them. Furthermore, from a social-psychological perspective, we also look into the students’ perceptions of self-identity and sense of belonging while studying in cross-boundary programs and how they cope with the cultural differences to re-generate identity and a sense of belonging.

## Methods

The empirical work of this study was conducted with a group of 28 students from mainland China studying higher diploma programs in one of Hong Kong’s largest vocational training institutions. Interviews were also conducted with teachers to draw views on how to support and cater to the students’ needs.

## Results

The findings suggest that in general, all students expected a multi-cultural learning experience, and they would like out-of-class connections and communication with teachers and classmates. Rapport building, interaction and mutual support with peers were also their requirements for better learning and mingling with local students. However, they had to overcome obstacles in their daily lives such as the language barrier, food and climate, and homesick to accommodate the local culture. They also showed awareness of their identity when studying in transnational and overseas programs. This further indicated that they constantly exercised self-actualization and neutralization activities to build friendship between local and non-local peers to blend into the local Hong Kong communities.

## Conclusions

Several implications were drawn from the findings of this study. First, to benefit cross-cultural understandings and enhance learning experiences, a tighter bonding between the local students, teachers and the students from mainland China is needed to nurture mutual acceptance. Secondly, the internationalization of the curriculum would enrich the programs with multi-perspective contents. In addition, the sense of belonging and identification with the cross-boundary programs of students from mainland China have yet to be enhanced to minimize the cultural differences. Given the small number of participants in this study, future research should include a larger sample size from different programs offered by different institutions for comparison.
